# Stearoyl-CoA Desaturase 1 as a Therapeutic Biomarker: Focusing on Cancer Stem Cells

**DOI:** 10.3390/ijms24108951

**Published:** 2023-05-18

**Authors:** Jin-Young Min, Do-Hee Kim

**Affiliations:** Department of Chemistry, College of Convergence and Integrated Science, Kyonggi University, Suwon 16227, Gyeonggi-do, Republic of Korea

**Keywords:** cancer stem cells, SCD1, phytochemicals, lipid metabolism, cancer progression

## Abstract

The dysregulation of lipid metabolism and alterations in the ratio of monounsaturated fatty acids (MUFAs) to saturated fatty acids (SFAs) have been implicated in cancer progression and stemness. Stearoyl-CoA desaturase 1 (SCD1), an enzyme involved in lipid desaturation, is crucial in regulating this ratio and has been identified as an important regulator of cancer cell survival and progression. SCD1 converts SFAs into MUFAs and is important for maintaining membrane fluidity, cellular signaling, and gene expression. Many malignancies, including cancer stem cells, have been reported to exhibit high expression of SCD1. Therefore, targeting SCD1 may provide a novel therapeutic strategy for cancer treatment. In addition, the involvement of SCD1 in cancer stem cells has been observed in various types of cancer. Some natural products have the potential to inhibit SCD1 expression/activity, thereby suppressing cancer cell survival and self-renewal activity.

## 1. Introduction

Tumor emergence and progression is an intricate process influenced by various factors. Within the tumor microenvironment, the self-renewal capacity of a tumor relies on a small subset of cells known as tumor-initiating cells and cancer stem cells (CSCs). These cells possess the ability to proliferate, undergo self-renewal, and are frequently associated with the reemergence of tumors following cancer treatment [1]. CSCs adapt their metabolism to facilitate tumor growth. To maintain the function of CSCs, they undergo crucial metabolic shifts such as dysregulation of glycolysis and lipid metabolism. CSCs hold a crucial position in driving the tumor progression, metastasis, infiltration, resistance to drugs, and relapse of cancer cells [2]. Lipid dysfunctions are associated with more aggressive molecular characteristics and increased transcripts related to lipogenesis and cholesterol synthesis pathways, which are linked to unfavorable survival outcomes [3]. Recent studies have been presented suggesting that disruptions in lipid metabolism, caused by the high need for energy and structural components, may have a significant impact on CSCs [4,5].

To maintain energy production, tumor cells need to regulate their nutrient intake and metabolism by reprogramming their metabolic pathways. Lipids play a critical role as a source of energy, structural components of biological membranes, signaling molecules, and regulators of redox homeostasis in cancer cells [6,7]. The dysregulation of lipid metabolism is affected by enzymes and signaling molecules that are involved in the lipid metabolism process. Lipid metabolism can modify the composition of the cell membrane, affect gene expression, and impact downstream signaling pathways that regulate various cellular processes, such as cell proliferation, motility, inflammation, and survival [8]. Lipids are an essential component of cells and organelle membranes, and fatty acids are also necessary for the proliferation of bulk tumor mass and the maintenance of cancer stem cells [9,10]. Alterations in both lipid catabolism and anabolism contribute to acquiring stemness characteristics in cancer cells, including lipid uptake, *de novo* lipogenesis, lipid desaturation, lipolysis, and fatty acid oxidation [11]. Recent reports implicate lipid desaturation as an essential process for cancer cell survival.

The lipid composition of cellular membranes affects membrane fluidity, cellular signaling, and, consequently, gene expression. Cancer cells have distinctive metabolic phenotypes that are characterized by the altered ratio of saturated to monounsaturated fatty acids (MUFAs) [12,13]. A critical committed step in the biosynthetic pathway of MUFAs is the introduction of the *cis* double bond between carbons 9 and 10 (Δ-9 position). The endoplasmic reticulum (ER) enzyme stearoyl-coenzyme A desaturase 1 (SCD1) converts saturated fatty acids (SFAs) into MUFAs, hence regulating the ratio of MUFAs to SFAs in cells [14]. This review highlights the role of SCD1, which is essential in the process of lipid desaturation in cancer cells and cancer stem cells [15,16].

## 2. Structure and Function of SCD1

The crystal structures of SCD1 in both mouse and human species were reported previously [17,18]. Mouse SCD1 shares 84% of its sequence identity with that of humans. SCD1 is an iron-containing transmembrane enzyme that consists of a cone-shaped structure formed by four α-helices. The protein is exclusively located on the ER membrane, with both the N- and C-terminal domains extending into the cytosol. Highly conserved regions surround the iron-containing center, including the eight histidines found in the cytoplasmic loop and C-terminus [17,19,20].

SCD is an integral membrane protein anchored to the ER membrane, where it catalyzes the biosynthesis of MUFAs from dietary or de novo synthesized SFA precursors. Four mouse SCD isoforms (SCD1-4) [21,22,23,24] and two human isoforms (SCD1 and 5) have been identified [25,26]. Two human SCD isoforms share the same enzymatic function but exhibit different tissue distribution patterns. SCD1 is found in almost all tissues, with major levels found in lipogenic tissues such as the liver and adipose tissue [27]; SCD5 is mainly expressed in the brain [28]. SCD1 plays a crucial role in lipid synthesis and the regulation of energy metabolism. SCD1 especially converts stearoyl-CoA (18:0) and palmitoyl-CoA (16:0) into the MUFAs oleoyl-CoA (18:1) and palmitoleoyl-CoA (16:1) through the insertion of a double bond in the Δ-9 position of the substrate [29]. These MUFA products of SCD1 desaturation are used as significant substrates for synthesizing a variety of complex lipids, including phospholipids, triglycerides, cholesteryl esters, wax esters, and other lipid species. SCD1 contributes to the fatty acid composition and fluidity of the membrane, influencing membrane-mediated biological signal transduction for the regulation of cell growth and differentiation [13,30]. It is also critically important in lipid metabolism and energy balance for storing or oxidation of lipids [31]. High SCD1 activity and/or expression has been found in a wide range of diseases, including atherosclerosis [32,33], obesity [34,35], and cancer [36,37]. In particular, reports that the expression and activity of SCD1 can play a key role in the pathogenesis of cancer have been attracting attention. In addition, there is mounting evidence indicating the potential value of SCD1 as a target for novel pharmacological approaches in cancer therapy [12,38,39,40,41,42,43].

## 3. Role of SCD1 in Cancer

Many studies have demonstrated the relevance of a supporting role for SCD1 in cancer progression of lung, breast, and prostate carcinomas, as well as clear cell renal cell carcinoma (ccRCC) [39,40,43,44,45,46]. The importance of SCD1 in cancer has already been reported in several review articles [12,38,47]. An increased ratio of MUFA/SFA propelled by high SCD1 expression appears to be a marker for the onset of typical traits of malignant behavior such as a high rate of cell proliferation, survival, and invasiveness [47]. It is recognized as a factor contributing to the lipogenic metabolism of cancer cells, followed by the biosynthesis of membrane phospholipids and energy-storage lipids [47]. In lung cancer, the ablation of SCD1 expression reduced the proliferation and invasiveness of cancer cells, consequently impairing tumorigenic capacity [39,48,49]. In addition, treating colon cancer cells with SCD1 inhibitors interrupted the cellular conversion of stearate to oleate in colon cancer cells, resulting in delayed tumor growth [46]. Hypoxia-inducible factor (HIF) is a well-known signaling mechanism that regulates SCD1 activity. In ccRCC, SCD1 was upregulated in response to HIF-2α. Increased SCD1 provides a sufficient substrate for lipid biosynthesis, which is required for rapid cell division. In addition, the upregulation of SCD1 also enhanced the expression of HIF-2α in feedback regulation by cooperating with HIF-1α, ultimately promoting tumorigenesis [50].

Recently, it has been demonstrated that inhibition of SCD1 in tumor cells can have antitumor effects by regulating immune cells in cancer tissues. Administering the SCD1 inhibitor A93957223 in mouse tumor models has been found to enhance antitumor T cells by recruiting dendritic cells (DCs) into tumors. SCD1 inhibition enhanced the production of chemokine C-C motif ligand 4 (CCL4) in cancer cells by reducing Wnt/β-catenin signaling. In addition, inhibiting SCD1 has been found to directly recruit DCs and induce CD8^+^ T cells induction, leading to increased production of CCL4 due to reduced ER stress [51]. The combination of SCD1 inhibition and anti-PD-1 therapy resulted in a synergistic effect, suggesting that targeting SCD1 may be a promising strategy for enhancing immunotherapy in cancer treatment [51].

The correlation between autophagy and SCD1 expression is also of increasing interest. Huang and colleagues demonstrated that pharmacological inhibition of SCD1 using CAY10566 induces autophagy-dependent apoptosis in human hepatocellular carcinoma (HCC) cells [52]. In addition, treatment involving the use of MF438 to inhibit SCD1 in lung cancer spheroid cells resulted in the activation of the ER stress response accompanied by a significant increase in autophagy, as determined by elevated levels of LC3-II [53]. Blocking SCD1 activity reversed the resistance of lung cancer sphere-forming cells to cisplatin [53]. Autophagy can act as both a cell survival mechanism and a tumor suppressor during tumorigenesis, depending on the context [54]. Ono et al. discovered that inhibiting SCD1 using both the small molecule T-3764518 and SCD1 shRNA in colon cancer HCT-116 cells accelerated the autophagic process and activated AMPK, enabling the cancer cells to escape the cytotoxic effects of SCD1 inhibition [55]. The inhibition of SCD1, leading to the excessive accumulation of saturated fatty acids, can activate the AMP-activated protein kinase (AMPK)-mediated resistance mechanism in HCT-116 cells. Therefore, when cells are exposed to both SCD1 and autophagy inhibitors, the activation of autophagy, known as a survival signal, is inhibited, ultimately resulting in the induction of cancer cell death [55]. In accordance with a previous report, the combination of amodiaquine treatment with SCD1 inhibition using A930572 showed a strong synergistic effect in inhibiting cancer cell proliferation, as demonstrated in lung cancer cells and a mouse xenograft model [56].

The importance of SCD1 function in cancer stem cells as well as cancer has been increasingly highlighted.

## 4. Role of SCD1 in Cancer Stem Cells

**Figure 1:**
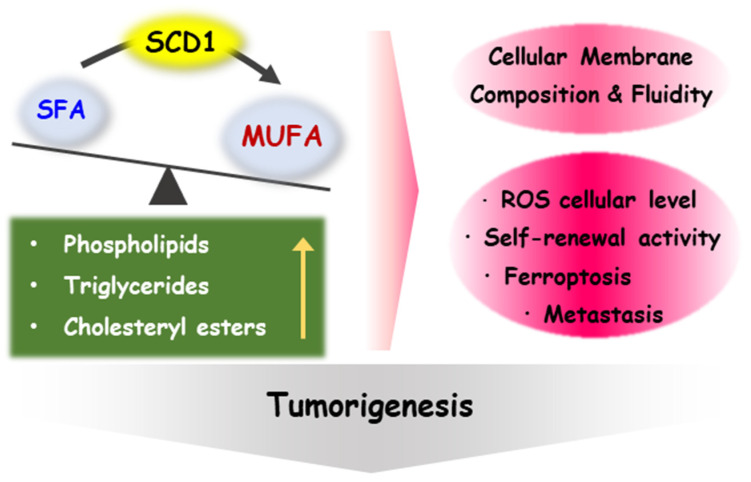
Role of SCD1 in tumorigenesis. MUFAs, as the products of SCD1 desaturation, are used as the building blocks for various types of complex lipids, such as phospholipids, triglyceride, cholesteryl esters, and other types of lipids. Fatty acid composition and the fluidity of the cell membrane are influenced by this factor, which contributes to the regulation of self-renewal activity, ferroptosis, and metastasis by affecting membrane-mediated biological signal transduction. It is also related to the regulation of ROS cellular levels.

Studies have suggested that CSCs have a higher proportion of MUFAs in their lipid composition compared to non-stem cancer cells, indicating that lipid desaturation could serve as a potential biomarker for CSCs in certain types of tumors, such as ovarian and glioblastoma. [15,57,58]. Membrane fluidity is defined as the degree of the molecular order and motion of membrane constituents, which is dependent on the content of unsaturated lipids [59]. Several studies have demonstrated that reducing membrane fluidity through anti-metastasis drugs can inhibit the metastatic capacity and stemness characteristics of breast cancer cells. This is thought to occur due to changes in membrane properties that affect signaling pathways and gene expression involved in these cellular processes [60]. In addition, Song et al. reported that MUFAs are required for the sphere formation of glioblastoma multiforme cell lines, as assessed by lipidomic profile differences between CSCs and bulk cancer cells [58]. MUFAs such as oleic acid, phosphatidylcholine, and phosphatidylethanolamine are enriched in CSCs compared to their non-stem counterparts [58]. The role of SCD1 in cancer stem cells has expanded to various cancer types, particularly lung, ovarian, breast, and prostate cancer [15,16,53,61]. The possible impact of SCD1 on tumorigenesis is summarized in Figure 1.

### 4.1. Ovarian Cancer

As mentioned earlier, SCD1 is an enzyme responsible for the desaturation of SFAs to MUFAs, which leads to an increase in the ratio of MUFAs to SFAs. This ratio was also significantly increased in ovarian cancer COV362 and OVCAR5 cells grown as spheres [15]. It is reported that ALDH^+^/CD133^+^ cells isolated from COV362 cells possess phenotypic characteristics of cancer stem cells and have a higher level of MUFAs [15]. Inhibition of SCD1 using a small molecule inhibitor or siRNA significantly reduced lipid unsaturation levels in ovarian cancer spheroids and then suppressed sphere-forming ability with decreased levels of *ALDH1A1*, *Nanog*, *Sox2*, and *Oct-4* mRNA expression [15]. Nuclear factor kappaB (NF-κB) is reported to be the key signal molecule that regulates SCD1 in ovarian CSCs. When treated with dimethylamino parthenolide, an inhibitor of NF-κB, primary ovarian cancer spheres exhibit reduced levels of both lipid unsaturation and *SCD1* mRNA expression [15]. Notably, the mRNA expression of SCD1 was regulated by p65, which binds directly to its promoter region. Conversely, treating SCD1 inhibitors in primary ovarian cancer spheres led to the suppression of NF-κB transcriptional activity.

Spheroids derived from the malignant ascites of ovarian cancer cells exhibit aberrantly elevated expression of SCD1 and fatty acid desaturase 2 (FADS2), positively accelerating lipid metabolic activities [62]. Pharmaceutical inhibitors of SCD1 and FADS2 suppressed sphere formation ability, which was accompanied by a reduction in the expression levels of self-renewal-related markers such as Krüppel-like factor 4 (KLF4) and Bmi-1 [62]. In addition, overexpression of SCD1 or FADS2 led to upregulation of the mesenchymal marker vimentin and epithelial-to-mesenchymal transition (EMT) regulators such as ZEB1, SNAIL, and Slug in ovarian cancer cells [62]. Blocking SCD1/FADS2 contributed to increased cellular reactive oxygen species (ROS) and lipid peroxidation through downregulated glutathione peroxidase 4 (GPX4) and the reduced glutathione/oxidized glutathione (GSH/GSSG) ratio in ascites-derived ovarian cancer cells, thereby promoting ferroptosis [62]. Moreover, co-treatment with SCD1/FADS2-specific inhibitors and cisplatin disrupted the metastatic spindle morphology of ovarian cancer patient-derived organoids [62].

### 4.2. Lung Cancer

SCD1 is overexpressed in the spheroids of lung cancer NCI-H460 cells and primary tumor cells derived from the malignant pleural effusions of patients with lung adenocarcinoma compared with adherent cultures [63]. Silencing and pharmacological inhibition of SCD1 reduced sphere-forming efficiency and was accompanied by attenuated mRNA expression of stem cell markers such as *ALDH1A1*, *Nanog*, and *Oct4* [63]. Spheroids treated with the SCD1 inhibitor MF-438 exhibited features of cellular damage, such as cytoplasmic vacuolization, mitochondrial swelling, and apoptotic nuclei [63]. In addition, SCD1 enzymatic inhibition selectively induced apoptosis of ALDH1A1-positive cells. Cells generated from spheroids in the presence of MF-438 show strongly decreased tumorigenic potential with impaired expression of ALDH1A1 [63].

High SCD1 mRNA and protein levels are associated with tumor progression and poor survival in lung adenocarcinoma [53,64]. High *SCD1* expression combined with stemness markers such as *CD24*, *CD133*, *SOX2*, and *CD44* is related to lung adenocarcinoma patients with worse prognosis [53]. SCD1 expression has been found to be upregulated in lung cancer spheroids, which are enriched for lung cancer-initiating cells [65]. The increased amount of unsaturated fatty acids caused by SCD1 can act as a substrate for the enzyme porcupine, also known as a membrane-associated *O*-acyltransferase. Wnt ligand undergoes post-translational acylation, which is mediated by porcupine. Wnt ligand combined with the frizzled class receptor 4 (FZD4) receptor causes inactivation of the destruction complex, ultimately stabilizing β-catenin and YAP/TAZ protein activity [65]. Resistance to cisplatin in lung cancer cells was attenuated through treatment of SCD1 with the pharmacologic inhibitor MF-438, which was confirmed by a sphere-forming assay [53]. In addition, the ALDH1A1^high^ cells isolated from lung cancer spheroids showed higher expression of *SCD1* and *NANOG*, and this effect was abrogated by simultaneous co-treatment with an SCD1 inhibitor [53]. Moreover, SCD1 activity has been linked to the activation of YAP and TAZ through the Wnt/β-catenin axis, thus contributing to the survival and spread of lung cancer stem cells [65]. Silencing of SCD1 caused inhibition in the protein expression of YAP and TAZ, which are required for the spheroid formation of lung cancer cells [65].

Epidermal growth factor receptor (EGFR) directly binds to SCD1 and phosphorylates its Tyr55 residue, which maintains the stability of SCD1 protein and increases MUFA levels to facilitate lung cancer growth [66]. EGFR activation was positively correlated with SCD1 Tyr55 phosphorylation, SCD1 protein expression, and poor patient prognosis in non-small cell lung cancer (NSCLC) tissues [66].

### 4.3. Liver Cancer

SCD1 mRNA expression was increased in the tumor tissues of approximately 60% of HCC patients compared to their non-tumor counterparts [67]. SCD1 was significantly upregulated in established sorafenib-resistant Bel7402 and Huh7 HCC cell lines and patient-derived tumor xenografts (PDTX) [67]. These cell lines established with shRNA against SCD1 exhibited a decrease in the percentages of CD24 and CD47 markers. Knockdown of SCD1 using shRNA sensitized HCC cells to sorafenib through the induction of ER stress, which is mediated by an increased mRNA expression of *CHOP* and *Bip* [67]. Co-treatment with SSI-4 (an inhibitor of SCD1 enzymatic activity) and sorafenib by oral gavage resulted in a synergistic effect on tumor growth inhibition in a PDTX model [67]. Sphere-forming cultures of HCC-enriched subpopulations with stem-cell characteristics are maintained by peroxisome proliferator-activated receptor-gamma (PPARγ) activation, which upregulates SCD1 expression and induces transcriptional activity and nuclear accumulation of β-catenin [68]. Treatment with PPARγ-specific antagonists or SCD1 inhibitors effectively decreased the sphere-forming capacity of HCC Huh7 and Hep3B cells, resulting in the loss of CSC properties through reduced expression of CSC-related markers such as *EpCAM*, *CD133*, *CD24*, *KRT19*, and *ICAM1* [68].

Inhibition of lipid unsaturation using shRNA against SCD1 or chemical inhibitor CAY10566 suppressed the proliferation of HCC JHH7 cells grown under monolayer and sphere cultures, which is mediated by the downregulation of *MYCN* mRNA expression [69]. In addition, the content of unsaturated fatty acids was increased in MYCN^high^/EpCAM^+^ CSC-rich HCC cells. It has been known that induction of ER stress causes a loss of intestinal epithelial stemness [70], while its reduction enables the maintenance of functional hematopoietic stem cells [71]. Both MYCN^high^/EpCAM^+^ CSC-like HCC cells and CSC-rich spheroids showed downregulation of ER stress-induced *activating transcription factor 3* (*ATF3)* gene expression [69]. Treatment with the ER stress chemical inducer stimulated the expression of the *ATF3* gene while reducing the expression of *MYCN* in JHH7 cells [69]. Moreover, the expression of ER stress-inducible transcription factor ATF3 was downregulated in MYCN^high^ CSC-like HCC cells, which was rescued through treatment with acyclic retinoid as a modulator of lipid desaturation [69].

### 4.4. Skin Cancer

In the Human Protein Atlas database, SCD1 is highly expressed in oral and skin squamous carcinoma samples [72]. Runx1 is known to be essential for the growth and survival of human oral and skin squamous cell carcinoma (SCC) cell lines [73]. Runx1 levels affect the membrane fluidity of cultured keratinocytes and human SCC cell lines by regulating SCD1 activity and the concentration of its product, oleate [72]. SCD1 expression induced Wnt activation, which may promote the activation and proliferation of keratinocyte and hair germ cells [72].

### 4.5. Bladder Cancer

High levels of SCD mRNA and protein have been associated with poor prognosis in patients with bladder cancer. SCD1 expression was upregulated in bladder cancer tissue samples compared with adjacent non-tumor tissues [74]. Inhibition of SCD activity was capable of decreasing the migration and invasion abilities of bladder cancer cell lines [74]. Blockade of SCD1 activity caused cell cycle arrest in the G1/S phase through downregulation of cyclin D1, Rb, Cdk4, and Cdk6. Interestingly, while SCD1 inhibitor A939572 did not induce apoptosis in parental bladder cancer cells, the inhibitor significantly inhibited the growth of sphere-forming cells [74].

### 4.6. Colon Cancer

The expression of SCD1 protein and mRNA is highly increased in colon adenocarcinoma HCT-15, HCT-116, SW480, and HT-29 cells grown under CSC-enriched culture conditions [75]. The ratios of MUFAs to SFAs and the levels of unsaturated lipids were significantly decreased in HCT116 colon CSCs treated with CAY10566, an SCD1 inhibitor [76]. Treatment with CAY10566 inhibited the spheroid formation of CSCs, indicating that SCD1 activity was associated with the stemness and tumorigenicity of colon CSCs [76]. In addition, the ratios of MUFAs to SFAs were higher in CSCs compared to parent colon HCT116 cancer cells [76]. Another SCD1 inhibitor, MF-438, did not kill the colorectal CSC population cells but regulated the expression of CSC-related signaling genes such as *AXIN*, *LEF1*, and *Notch1* [77]. Moreover, irinotecan-resistant colon cancer cells led to a decrease in MUFAs with higher levels of SCD1 compared with their parental cells [78]. SCD1 directly regulates the expression of ALDH1A1, which is a CSC biomarker that can stimulate ROS generation and cancer stemness in irinotecan-resistant colon cancer cells [78].

### 4.7. Glioblastoma

Overexpression of sterol-regulated element-binding protein 1 (SREBP1) has been observed in glioblastoma multiforme (GBM), leading to enhanced lipid metabolism associated with abundant MUFAs [79]. SCD1 is one of the target genes regulated by SREBP1, and it has been shown to be required for tumor growth in several types of cancer [79]. Silencing of SCD1 reduced cell viability in patient-derived glioblastoma stem cells (GSCs), while there was no change in normal human astrocytes [80]. Ectopic expression of SCD1 led to a greater frequency of stem cells in GSCs and promoted cell growth and the formation of secondary spheres. Following intracranial transplantation of GSCs in mice, it was confirmed that more tumors developed in the mice group in which SCD1 was overexpressed [80]. Intranasal delivery of the SCD1 inhibitor CAY10566 into a PDTX mouse model showed the ability to inhibit tumor formation [80]. In addition, activation of SCD1 transcription and the subsequent synthesis of MUFAs plays a cytoprotective role in mitigating ER stress [80]. Using a dataset from The Cancer Genome Atlas, a positive correlation was observed between transcript levels of *BiP* and *SCD1* in the GBM patient group [80]. Inhibition of SCD1 led to the accumulation of SFAs, which in turn exacerbated ER stress. GSCs treated with the SCD1 inhibitor CAY compound displayed an increase in ER stress markers such as *Bip*, *ChOP*, *sXBP1*, and *GADD34* [80]. The activity of SCD1 provides GSCs with a survival advantage, making them vulnerable to metabolic targeting via SCD1 inhibition [80].

### 4.8. Gastric Cancer

SCD1 has been shown to enhance the population of gastric cancer sphere cells through the Hippo/YAP pathway [81]. The use of an inhibitor or siRNA to suppress SCD1 reduced the expression of stemness-associated cell surface markers such as CD44, CD133, and Lgr5 and transcriptional levels of *Sox-2*, *Oct-4*, and *Nanog*. This impairs the sphere-forming ability of patient-derived gastric cancer cells [81]. Inhibition of SCD1 led to disassembly of YAP in the nucleus via decreased YAP phosphorylation, which attenuates the expression of TEA domain family member 1 (TEAD1) and cyclin D1 [81]. In addition, when cells treated with A939572, a pharmacological inhibitor of SCD1, were injected subcutaneously into SCID mice, the ability to form tumors was significantly suppressed [81].

### 4.9. Breast Cancer

SCD1 is overexpressed and correlates with poor prognosis in breast cancer patients [82]. CD10 is known to degenerate osteogenic growth peptide (OGP), which is recognized as an anti-tumoral peptide, and is expressed in cancer-associated fibroblasts to support tumor stemness and induce chemoresistance [83]. CD10 sustains the characteristics of cancer stem cells by cleaving the active domain of OGP in mammosphere formation [83]. In addition, OGP treatment of MCF-7 mammosphere cells reduced SCD1 expression and subsequently impaired lipid desaturation [83].

Table 1 provides a summary of the consequences of inhibiting SCD1 in cancer stem cells of various types.

## 5. Regulation of SCD1 Expression by Anti-Carcinogenic Natural Compounds

### 5.1. Betulinic Acid

Betulinic acid (BetA), a lupine-type pentacyclic triterpenoid saponin from tree bark, is known to exhibit cytotoxicity against various cancer cells but not normal cells [84,85]. BetA inhibited the activity of SCD1, which regulates the saturation level of cardiolipin, a lipid known for mitochondrial structural function [86]. BetA caused an increase in cardiolipin saturation within the mitochondria membrane, resulting in the release of cytochrome c and triggering apoptosis in HeLa cells. Under the same experimental condition, this was also confirmed by SCD1 knockdown using siRNA [86]. In addition, BetA inhibited the proliferation of gallbladder cancer NOZ cells by disturbing mitochondrial membrane potential [87]. Additionally, BetA suppressed SCD1 expression at both the mRNA and protein levels in NOZ cells. Administration of BetA decreased tumor size in xenograft mice injected with NOZ cells, and SCD1 expression was also suppressed [87]. Moreover, in primary colorectal cancer stem cells, betulinic acid induced apoptosis as well as a loss of clonogenicity, which is caused by SCD1 inhibition [88].

### 5.2. Curcumin

Curcumin is a dietary polyphenol compound derived from rhizomes of turmeric (*curcuma longa*). It has been shown to have anti-cancer effects in *in vitro*, *in vivo*, and pre-clinical studies [89]. Curcumin inhibited primary and secondary mammosphere formation based on breast cancer MCF-7 and SUM149 cells. In addition, curcumin treatment resulted in downregulated expression of SCD1 in ALDH^+^ populations sorted from primary breast cells [90]. Moreover, the regulatory effects of curcumin on lipogenic enzymes have attracted increasing attention as a potential means of inhibiting or reversing tumor growth [90].

### 5.3. Trichothecin

Trichothecin is a sesquiterpenoid isolated from the endophytic fungus of the herbal plant *Maytenus hookeri* Loes. SCD1, which is highly expressed in aggressive colorectal carcinoma, was attenuated by trichothecin treatment and restored saturated fatty acid levels [91]. In addition, the transcriptional activity of the SCD1 promoter region was reduced through trichothecin treatment in colon cancer LOVO and HCT116 cells, contributing to the anti-invasive effect [91]. It can be suggested that the anti-tumor effects of trichothecin may be related to SCD1-mediated fatty acid metabolite alterations.

### 5.4. Icaritin

Icaritin is a prenylflavonoid derivative from the Chinese herbal medicine *Eimedii Herba* and has been known to exert anti-cancer effects. The possibility of the direct binding of icaritin to SCD1 is suggested by computer-aided drug design [82]. Icaritin inhibited the growth of MCF-7 and SK-BR-3 cells in the mitochondrial pathway by reducing the expression and activity of SCD1 [82]. An icaritin derivative induced autophagy through AMPK/mTOR and mitogen-activated protein kinase signaling pathways in MCF-7 cells. Icaritin-induced autophagy was alleviated by overexpression of SCD1 in MCF-7 cells.

## 6. Concluding Remarks and Future Perspectives

**Figure 2:**
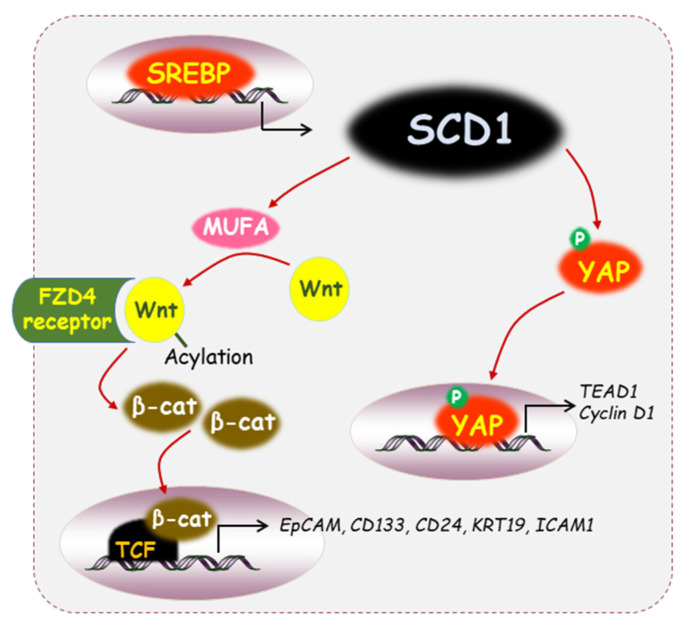
Diverse signaling mechanisms through SCD1 in cancer stem cells. SCD1 is one of the target genes regulated by SREBP1. Decreased YAP phosphorylation resulting from SCD1 inhibition caused the disassembly of YAP in the nucleus, leading to a reduction in the expression of TEAD1 and cyclin D1. Post-translational acylation of the Wnt ligand, which MUFA mediates, enables its binding with the FZD4 receptor, resulting in the stabilization of β-catenin and leading to the expression of CSC regulatory genes.

High SCD1 expression has been reported in many malignancies, including cancer stem cells [15,16,53,61]. Some dietary and natural anti-cancer compounds regulate the expression/activity of the SCD1 gene/protein, which results in restoration of the proportion of SFAs. Downregulation of SCD1 expression is associated with inhibited proliferation, migration, metastasis, and growth of cancer (stem) cells. However, the molecular mechanisms underlying restoration of SCD1 function by natural anti-cancer compounds remain largely unknown. Several key signaling mechanisms involving SCD1 in cancer stem cells are presented in Figure 2, which are expected to have the potential to lead to the discovery of phytochemicals or synthetic inhibitors. Our research team achieved experimental results indicating that thymoquinone can act as a growth inhibitor by inhibiting the expression of SCD1 in breast cancer cells, and related research is still in progress.

It becomes particularly interesting to investigate the role of SCD1 in cancer (stem) cells, which is associated with obesity, lipid accumulation, and inflammation [92,93]. In addition, tumorigenesis is linked to insulin-resistant glucose metabolism [94]. Under the condition of insulin resistance, expression of glucose transporter type 4 is blocked, resulting in elevated blood glucose levels and increased β-oxidation of fatty acids [95]. As SCD1 is linked with insulin resistance in morbidly obese patients [96], SCD1 may serve as a connection in the association between insulin resistance and cancer.

High SCD1 expression is a major cause of the increased ratio of MUFAs/SFAs, which contributes to the fatty acid composition and fluidity of the membrane. This influences membrane-mediated biological signal transduction to regulate cell growth and differentiation [13,30]. Cellular membrane composition of the MUFA/SFA ratio can be modulated by SCD1 and is crucial for lipid metabolism and energy balance for the storage or oxidation of lipids. Reducing membrane fluidity using anti-metastasis drugs in cancer cells inhibits metastatic capacity and stemness characteristics. Downregulation of SCD1 expression in tumor cells is a reliable therapeutic strategy to treat cancer. Therefore, natural anti-cancer compounds may contribute to maintaining cellular membrane status through the regulation of SCD1 expression or activity, which could be an alternative therapeutic target for chemoresistant cancer cells or cancer stem cells.

## Figures and Tables

**Table 1:** The effects of inhibiting SCD1 on different types of cancer cell lines.

Cell Type	Regulated Genes or Proteins	Phenotypic Effects	Ref.
COV362, OVCAR5	ALDH1A1, Nanog, Sox2, Oct-4↓	Sphere-forming ability↓	[15]
Ascites-derived ovarian cancer cells	KLF4↓, ROS↑, GPX4↓, GSH/GSSG ratio↓	Ferroptosis↑, EMT↓, sphere-forming ability↓	[62]
NCI-H460	ALDH1A1, Nanog, Oct-4↓	Sphere-forming ability↓	[63]
Patient-derived lung cancer tissue	YAP, TAZ activity↓	Sphere-forming ability↓	[65]
Sorafenib-resistant Bel7402 and Huh7 cells	CD24, CD47↓ CHOP, Bip↑	ER stress↑, sphere-forming ability↓	[67]
Huh7 and Hep3B cells	EpCAM, CD133, CD24, KRT19, ICAM1↓	Sphere-forming ability↓	[68]
JHH7 cells	ATF3, MYCN↓	Sphere-forming ability↓	[69]
UMUC3 and RT4 cells	Cyclin D1, Rb, Cdk4, Cdk6↓	Cell cycle arrest↑, migration and invasion↓, sphere-forming ability↓	[74]
Patient-derived glioblastoma stem cells	Bip, ChOP, sXBP1, GADD34↑	Cell viability↓ sphere-forming ability↓	[80]
Gastric cancer stem-like HSC034 cells	Sox2, Oct4, Nanog, CD44, Lgr5, CD133↓, YAP, TEAD1↓	Sphere-forming ability↓, metastasis↓	[81]

## Data Availability

Not applicable.

## Abbreviations

AMPK, AMP-activated protein kinase; ATF3, activating transcription factor 3; CCL4, chemokine C-C motif ligand 4; ccRCC, clear cell renal cell carcinoma; CSCs, cancer stem cells; DCs, dendritic cells; ER, endoplasmic reticulum; EGFR, epidermal growth factor receptor; FADS2, fatty acid desaturase 2; FZD4, frizzled class receptor 4; GBM, glioblastoma multiforme; GSH/GSSG, reduced glutathione/oxidized glutathione; GPX4, glutathione peroxidase 4; HCC, hepatocellular carcinoma; HIF, hypoxia-inducible factor; KLF4, Krüppel-like factor 4; MUFAs, monounsaturated fatty acids; NF-κB, nuclear factor kappaB; OGP, osteogenic growth peptide; PDTX, patient-derived tumor xenografts; PPARγ, proliferator-activated receptor-gamma; ROS, reactive oxygen species; SCC, squamous cell carcinoma; SCD1, stearoyl-coenzyme A desaturase 1; SFAs, saturated fatty acids; SREBP1, sterol-regulated element-binding protein 1; TEAD1, TEA domain family member 1.
